# New Constituents from the Korean Sponge *Plakortis simplex*

**DOI:** 10.3390/md11114407

**Published:** 2013-11-05

**Authors:** Jung Soon Oh, Buyng Su Hwang, Ok-Hwa Kang, Dong-Yeul Kwon, Jung-Rae Rho

**Affiliations:** 1 Department of Oceanography, Kunsan National University, Jeonbuk 573-701, Korea; E-Mails: jsoh@kunsan.ac.kr (J.S.O.); sybsl@naver.com (B.S.H.); 2 College of Pharmacy and Wonkwang-Oriental Medicines Research Institute, Institute of Biotechnology, Wonkwang University, Jeonbuk 570-749, Korea; E-Mails: kangokhwa@naver.com (O.-H.K.); sssimi@wku.ac.kr (D.-Y.K.)

**Keywords:** *Plakortis simplex*, cyclic peroxide, cyclic peroxyketal, pyridinium, NMR

## Abstract

Six new cyclic peroxides (**1**–**6**) were isolated from the Korean sponge *Plakortis simplex*, along with two new alkylpyridinium alkaloids (**7** and **8**). The structures of these compounds were completely determined by a combination of NMR analysis and chemical reactions. Compounds **1**–**6** exhibited cytotoxic/antifungal activities against RAW264.7 cells and *Candida albicans*.

## 1. Introduction

Sponges of the family Plakinidae have provided structurally noble and biologically active metabolites including alkaloids, glycolipids, lactones and polyketides [1,2,3,4,5]. In particular, numerous polyketides containing a five- or six-membered peroxide group have been isolated from Plakinidae since the identification of plakortin in 1978 [6,7,8,9,10]. These compounds also exhibited several biological activities, such as cytotoxic, antifungal, and antimalarial properties, making them potentially useful in the development of drugs or as research tools [11,12,13].

As part of our search for new metabolites from the extracts of the Korean sponges, we isolated six cyclic peroxides (**1**–**6**) from fractions of *Plakortis simplex* which showed toxicity against *C. albicans* in a disk agar diffusion method. Additionally, two new alkaloids (**7** and **8**) were isolated from the more polar fraction. All compounds had a common straight chain composed of ten carbons. In particular, compounds **2**–**6** contained the identical side chain, but the substituents and configurations on a 1,2-dioxane ring were dissimilar between the five compounds. Compounds **2**–**4** were characterized as peroxyketals with a methoxy group on C-6, predominantly isolated from Indonesian sponges [14,15], and compounds **5** and **6** as cyclic peroxides with a methyl group on C-6, frequently found in Caribbean sponges [16,17]. The isolation of these two different types of peroxides from the same extract is interesting and, to the best of our knowledge, the first example in marine sponges. New alkaloids **7** and **8**, which were isolated from the same extract, were found to possess the same skeleton as platisidines A and B [18], although the length of the linear chain varied.

Herein, we describe the isolation, stereostructural characterization of all of the isolated compounds, together with activities for six peroxide compounds.

## 2. Results and Discussion

The methanolic extract of *P. simplex* was partitioned between H_2_O and dichloromethane, and the organic layer was subjected to reversed-phase silica flash chromatography, eluting with a stepwise gradient from 50% to 100% MeOH in H_2_O at 10% MeOH increments to yield a total of six fractions. Of the fraction obtained, the 90% MeOH fraction exhibiting relatively wider zones of inhibition in a disk diffusion assay was further separated by repeated HPLC to afford compounds **1**–**6**. The isolated peroxide carboxylic acids were easily decomposed at room temperature and in CDCl_3_ solvent, yet sustained in CD_3_OD solvent for NMR analysis and activity tests for a few days. Additionally, alkaloids **7** and **8** could be isolated from the more polar fraction (70% MeOH) showing ^1^H NMR signals in the aromatic region.

Compound **1** was determined to have a molecular formula of C_18_H_32_O_5_ on the basis of high-resolution fast-atom bombardment mass spectrometry (HRFABMS) and the ^13^C NMR spectrum, which was consistent with three degrees of unsaturation. The IR spectrum exhibited the presence of a carbonyl and a hydroxyl group from the characteristic absorption bands at 1457, 1714, and 2926 cm^−1^. The ^1^H NMR spectrum of compound **1** in CD_3_OD showed comparatively few resonances, including an overlapping signal corresponding to an aliphatic saturated chain and a singlet at δ_H_ 3.22. The ^13^C NMR and the edited heteronuclear single quantum coherence (HSQC) spectra were assigned to two methyls, one methoxy, nine upfield-shifted methylenes, one methine, one oxymethine, two olefinic carbons, one carboxy (δ_C_ 175.5) and one unusual downfield-shifted quaternary carbon (δ_C_ 104.3). Moreover, the above functionalities and the remaining one degree of unsaturation indicated the presence of a ring in this compound.

**Figure 1:**
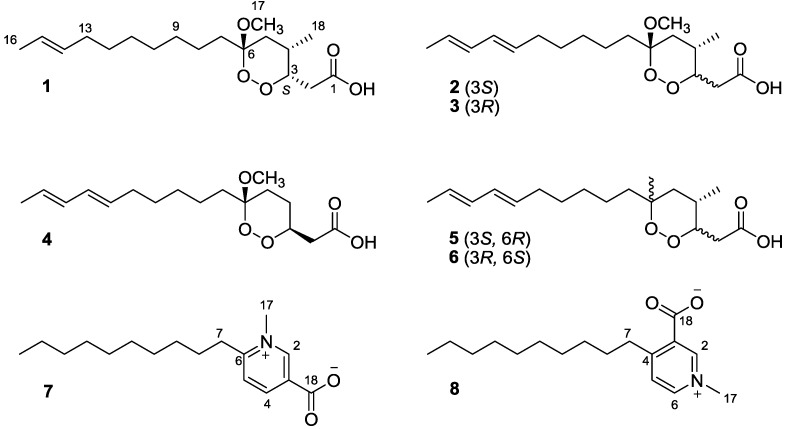
The chemical structures of compounds **1**–**8**.

Careful examination of correlation spectroscopy (COSY) and HSQC correlations revealed a methyl-branched carbon chain from C-2 to C-5 with two pairs of geminal coupling protons. Although the signals around δ_H_ 2.47 in the ^1^H NMR spectrum were overlapping, the structure of the fragment was confirmed by the related HSQC and heteronuclear multiple-bond correlation (HMBC) correlations. Further HMBC correlations showed a connection of the carboxy group to C-2 and of the quaternary carbon at δ_C_ 104.3 to C-5, and a methoxy group at the quaternary carbon C-6 (Figure 1). In this partial structure, the carbon chemical shifts of C-3 and C-6 and two unassigned oxygens introduced a 1,2-dioxane ring via a peroxide linkage between the only oxygen-bearing carbons C-3 and C-6, which also accounted for the one degree of unsaturation mentioned above. Other unassigned ^1^H and ^13^C NMR resonances were assigned to characterize one linear aliphatic chain, one olefinic methyl, and one double bond, which were combined to form a dec-2-enyl unit. Finally, the attachment of this unit to the C-6 quaternary carbon from the HMBC correlation of H-7/C-6 completed the planar structure of **1**.

**Figure 2:**
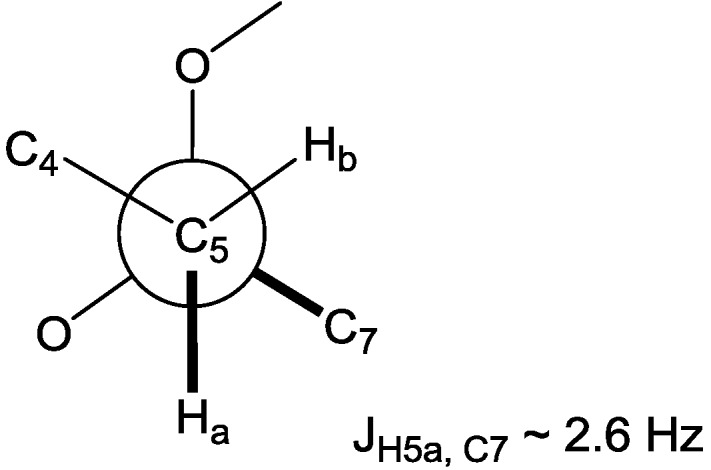
Newman projection for C-4 ~ C-7 representing the *gauche* orientation between H-5a and C-7.

**Figure 3:**
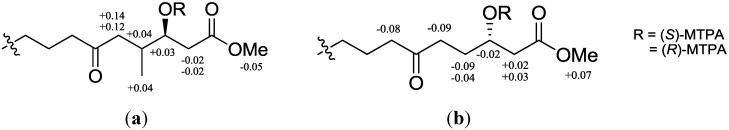
^1^H NMR chemical shift differences (Δδ*^SR^*) in CDCl_3_ of (**a**) (*S*/*R*)-MTPA esters after methyl ester and reductive cleavage of 1,2-dioxane of compound **1**; (**b**) **4**.

The double bond in the side chain could be assigned as *E* configuration by the downfield shifted che mical shift (about 33 ppm) of the allylic carbon C-13 [19]. The configurational assignments of three chiral centers in the 1,2-dioxane ring were determined on the basis of the NOE correlations and the well-resolved spin coupling constants. First, the splitting pattern of H-3 was analyzed to give three doublet coupling constants of 9.3, 3.9, and 3.9 Hz, indicating spin couplings with one axial and two equatorial protons. Next, the H-5a proton triplet, which is in an axial position with H-4, showed the NOE correlations with both H-2b and the methyl proton Me-18. These two observations enabled us to assign the relative configurations of C-3 and C-4 as 3S* and 4S*, respectively. On the other hand, the methoxy methyl protons on C-6 had a strong NOE correlation with the H-7 methylene protons, yet this did not enable assignment of the configuration of C-6. Instead, H-5a showed a long range coupling with the methoxy methyl carbon C-17, resulting from the *W* coupling, in the HMBC spectrum. Furthermore, the configuration of C-6 could be supported by a small heteronuclear coupling constant (^3^*J*_H5a,C7_ ~ 2.6 Hz) for the *gauche* orientation between H-5a and C-7 in the heteronuclear single quantum multiple bond correlation (HSQMBC) experiment, assigning C-6 as 6R* (Figure 2). The absolute stereochemistry of **1** was established based on the modified Mosher method on C-3 after methylation with diazomethane and the reductive cleavage reaction of the 1,2-dioxane ring [14]. The difference of chemical shifts in the ^1^H NMR spectra of the (*S*/*R*)-MTPA esters indicated the *S* configuration at C-3, as shown in Figure 3a. Therefore, compound **1** was determined as 2-((3*S*, 4*S*, 6*R*)-6-((*E*)-dec-8-enyl)-6-methoxy-4-methyl-1,2-dioxan-3-yl) acetic acid.

HRFABMS revealed compounds **2** and **3** to have the same molecular formula C_18_H_30_O_5_, indicative of four degrees of unsaturation. Two compounds contained a diene group based on the NMR signals for two double bonds and UV absorption at 228 nm, but were dissimilar in the ^1^H and ^13^C NMR spectra. In particular, the ^1^H and ^13^C chemical shifts of C-2 to C-4 in **2** were highly similar to those of compound **1**, while those of **3** were shifted to the upfield region. Analysis of 2D NMR data for compounds **2** and **3** showed their planar structures to be identical, suggesting that these compounds are stereoisomers. Although the number of double bonds is different, the high degree of similarity between the ^1^H and ^13^C chemical shifts of compound **2** and those of **1** demonstrated that the configurations of three chiral centers on the 1,2-dioxane ring of compounds **1** and **2** are identical. In compound **3**, the H-3 oxymethine proton exhibited the splitting pattern with three doublet coupling constants of 3.2, 9.3, and 9.3 Hz, representing an equatorial coupling and two axial couplings with adjacent protons. More apparently, the NOE correlation between H-5a and H-3 revealed that the orientation of H-3 was opposite to that of **2**. The orientation of H-4 in **3** could also be determined as 4*S* by both the coupling constant (*J*_HH_ = 13.0, 4.4 Hz) for H-5b and the NOE cross peak between H-3 and 18-Me. As previously shown, the configuration of C-6 could be established as 6*R*. On the other hand, the diene group in compounds **2** and **3** was commonly placed next to the terminal methyl group appearing as an olefinic doublet, which was supported by their COSY correlations. Furthermore, the group was found as (*E*, *E*) configuration from the values of large coupling constants between H-12 and H-13, and H-14 and H-15, and NOE correlations of H-14/16-Me and H-11/H-13. Therefore, compound **3** was identified as the C-3 epimer of **2** and named 2-((3*R*, 4*S*, 6*R*)-6-((6*E*, 8*E*)-deca-6,8-dienyl)-6-methoxy-4-methyl-1,2-dioxan-3-yl) acetic acid.

HRFABMS revealed that compound **4** had a sodium adducted peak at *m/z* = 335.1841 for [M + Na]^+^, suggesting a molecular formula of C_17_H_28_O_5_. Compared to the ^1^H NMR spectra of the previous compounds, that of compound **4** was characteristic of the absence of a methyl doublet signal in the most upfield region. The planar structure of **4** resembled that of compounds **2** and **3**, with the exception of the loss of the methyl group at the position of C-4 by interpretation of 1D and 2D NMR spectra. This was recognized from the H-4 methylene proton coupled to the H-3 proton. Configurational assignments of two substituents on the 1,2-dioxane were assigned as 3*S* for C-3 and 6*R* for C-6 by the NOE correlations of H-5a with H-3 and H-7. The stereochemistry of C-3 and C-6 was also supported by the modified Mosher method (Figure 3b) and comparison of the carbon chemical shifts of C-6 and C-7 with those of **2**, respectively. Thus, compound **4** was named as 2-((3*S*, 6*R*)-6-((6*E*, 8*E*)-deca-6,8-dienyl)-6-methoxy-1,2-dioxan-3-yl) acetic acid.

**Figure 4:**
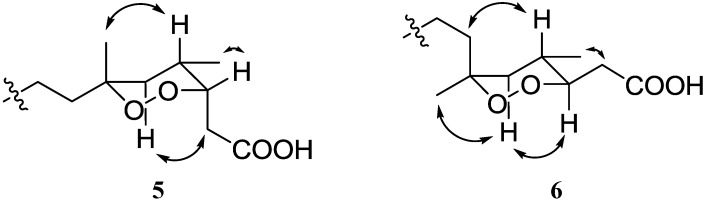
Key NOE correlations for compounds **5** and **6**.

The molecular formula of compound **5**, C_18_H_30_O_4_, was determined on the basis of the HRFABMS analysis, and it was identical to that of **6**. Compared to the previous compounds **1**–**4**, the ^1^H and ^13^C NMR spectra of the two compounds contained a methyl singlet in place of a methoxy signal at δ_H_ 3.22. Similarly, 1D and 2D NMR analysis suggested that the planar structures of compounds **5** and **6** were quite similar to those of compounds **2** and **3**, with the exception of the attachment of a methyl group on C-6. Moreover, the values of chemical shifts and coupling constants for C-2 to C-4 in **5** were very similar to the corresponding values in **2**, and the spectral values for C-2 to C-4 in **6** were to those in **3**. This observation was sufficient to designate the configuration of C-3 as 3*S* in compound **5** and 3*R* in **6**. Based on these different configurations, the C-4 stereochemistry of the two compounds could be explained as a common *S* form by analysis of each proton-proton coupling pattern for H-3. Interestingly, unlike the previous compounds, there was a clear difference in the ^1^H and ^13^C chemical shifts of C-6 to C-8 and Me-17 in compounds **5** and **6**, suggesting the different configurations on C-6. The configuration of C-6 in **5** was defined as *R* from both the NOE correlation between H-4 and Me-17 proton and the strong HMBC correlation between H-5a and Me-17 carbon. In contrast, compound **6** was characterized by the NOE correlation between H-5a and Me-17 and the strong HMBC correlation between H-5a and C-7, indicating the configuration of C-6 to be *S* as shown in Figure 4.

Accordingly, compounds **5** and **6** were designated as 2-((3*S*, 4*S*, 6*R*)-6-((6*E*, 8*E*)-deca-6,8-dienyl)-4,6-dimethyl-1,2-dioxan-3-yl) acetic acid and 2-((3*R*, 4*S*, 6*S*)-6-((6*E*, 8*E*)-deca-6,8-dienyl)-4,6-dimethyl-1,2-dioxan-3-yl) acetic acid, respectively.

Compounds **7** and **8** had the same molecular formula, C_17_H_27_NO_2_, which was determined on the basis of the sodium adduct [M + Na]^+^ in HRFABMS. These compounds significantly differed in their ^1^H NMR spectra, compared to that of compounds **1**–**6**. First, compounds **7** and **8** showed a common typical AMX pattern in the aromatic region and the upfield resonances corresponding to an aliphatic linear chain. Resonances in the ^13^C NMR spectra of both compounds were also assigned to one methyl, one *N*-methyl, three sp^2^ methines, two sp^2^ quaternary carbons and nine methylenes with the aid of the edited HSQC spectrum. Although not observed in the ^13^C NMR spectra of the two compounds, the presence of a carboxylate group was inferred from the unassigned molecular formula and an IR absorption band around 1650 cm^−1^. In compound **7**, the *N*-methyl singlet at δ_H_ 4.32 was correlated with two carbons at δ_C_ 148.6 (C-2) and 161.1(C-6) in the HMBC spectrum. Further HMBC correlations of H-2/C-4, H-2/C-6, H-4/C2, H-4/C-6, and H-5/C-3, along with a strong coupling between H-4 and H-5, defined an *N*-methyl pyridinium ring containing two substituents. One of the substituents was a decyl group, which was apparent from the carbon signals and the COSY correlations, and was placed at the position C-6 of the ring based on the HMBC cross peaks of H-7/C-5, H-7/C-6. The other is the carboxylate group linked to C-3, which was evidenced by the HMBC correlation of H-4 with the carbon at δ_C_ 159.5. Thus, the structure of **7** was revealed to be 6-decyl-1-*N*-methyl pyridinium-3-carboxylate. Analogously, COSY and HMBC spectral analysis of compound **8** revealed the same *N*-methyl pyridinium moiety as that in **7**, referred to as trigonelline. In this case, the decyl group was connected to C-3 of the trigonelline on the basis of the HMBC correlations of H-7/C-3 and H-7/C-5. Thus, compound **8** was designated as 4-decyl-1-*N*-methyl pyridinium-3-carboxylate. The structure for compounds **7** and **8** was very similar to that of platisidine A and B, respectively, which commonly contained *n*-hexadecyl side chain [18].

Compounds **1**–**6** showed considerable cytotoxicity toward RAW264.7 cells with EC_50_ values of 2–4 µg/mL. Compounds **1**–**6** also demonstrated strong activity in a disk diffusion assay against *C. albicans* (*ca.* 16–20 mm) better than for amphotericin (*ca.* 15 mm), which was used as a positive control. This may be due to the strong cytotoxicity rather than any sort of specific antifungal activity.

## 3. Experimental Section

### 3.1. General Experimental Procedures

Preparative HPLC was carried out on a Varian Prostar with a refractive index detector using a YMC H80 column (150 mm × 20 mm ID). Optical rotations were measured on a JASCO P-1010 polarimeter with a 5 cm cell. UV spectra were obtained in MeOH using a Varian Cary 50 and IR spectra were measured on a JASCO FT/IR 4100 spectrometer. All NMR spectra were recorded on a Varian VNMRS 500 spectrometer in CD_3_OD and the ^1^H NMR spectrum for the MTPA compounds in CDCl_3_ solvent. Chemical shifts of the proton and carbon spectra measured in CD_3_OD solution were reported in reference to residual solvent peaks at 3.30 ppm and 49.0 ppm, respectively. High resolution mass spectra were obtained on a JEOL JMS-700 spectrometer at the Korea Basic Science Research Institute, Daegu, Korea.

### 3.2. Material

The specimen of *Plakortis simplex* (Sample No. 08K-6) was collected by hand using SCUBA at a depth of 30 m in 2008 off the shore of Keomun Island at the West Sea of South Korea and identified by Dr. Chung J. Sim. The sponge is flat, measuring 75 mm × 55 mm wide and 15 mm thin. Texture is smooth and oscules are rare. In the skeleton, rare diods and triods are densely packed, measuring diods 70–125 × 2–4 µm and triods 60–95 × 3–4 µm. A voucher specimen (08K-6) is deposited at the Marine BioResources Bank, Hannam University, Daejeon, Korea.

### 3.3. Extraction and Isolation

The collected specimen was frozen on site and delivered to the laboratory under dry ice, and then kept in a refrigerator at −25 °C until study. Freshly thawed sponge was chopped into small pieces and extracted twice with MeOH at room temperature. The methanolic extract (*ca*. 20 g) was partitioned between dichloromethane and H_2_O solvents. The organic layer was evaporated under reduced pressure and repartitioned between *n*-hexane and 15% aqueous MeOH for defatting. Then the aqueous MeOH fraction (1.5 g) was subjected to reversed phase silica gel flash column chromatography eluting with solvents of decreasing polarity (MeOH/H_2_O = 50/50; 60/40; 70/30; 80/20; 90/10; 100% MeOH; 100% acetone) to give seven fractions. The fraction (400 mg) eluted with 90% MeOH showed the cytotoxicity on the brine shrimp lethality test and antifungal effect against *C. albicans* in a disk diffusion method. This fraction was first separated by reversed phase HPLC (YMC H80 column, 150 mm × 20 mm ID) eluting with 25% aqueous MeOH with the flow rate of 5 mL/min to give six compounds, **1**–**6**. Further purification of each compound was conducted by HPLC using analytical column (YMC H80, 150 mm × 4.6 mm ID) with 40% aqueous MeCN solvent system. In addition, compounds **7** and **8**, which showed different NMR signal pattern, were isolated from 70% MeOH fraction eluting with 40% aqueous MeOH solvent under the same HPLC column to get a small quantity of 2 mg each.

Compound **1**: white amorphous oil; [α]^25^_D_ −212 (*c* = 0.15, MeOH); IR (film) ν_max_ 2926, 1714, 1457, 1083 cm^−1^; ^1^H and ^13^C NMR data are given in Table 1 and Table 2, respectively; HRFABMS *m/z* 351.2149 (calcd for C_18_H_32_O_5_Na, 351.2147).

Compound **2**: white amorphous oil; [α]^25^_D_ −189 (*c* = 0.15, MeOH); UV (MeOH) λ_max_ (log ε) 228 (4.20) nm; IR (film) ν_max_ 3360, 2929, 1714, 1458, 1083 cm^−1^; ^1^H and ^13^C NMR data are given in Table 1 and Table 2, respectively; HRFABMS *m/z* 349.1986 (calcd for C_18_H_30_O_5_Na, 349.1991).

Compound **3**: white amorphous oil; [α]^25^_D_ −109 (*c* = 0.15, MeOH); UV (MeOH) λ_max_ (log ε) 228 (4.20) nm; IR (film) ν_max_ 2928, 1715, 1434, 1081 cm^−1^; ^1^H and ^13^C NMR data are given in Table 1 and Table 2, respectively; HRFABMS *m/z* 349.1984 (calcd for C_18_H_30_O_5_Na, 349.1991).

Compound **4**: white amorphous oil; [α]^25^_D_ −138 (*c* = 0.15, MeOH); UV (MeOH) λ_max_ (log ε) 227 (4.00) nm; IR (film) ν_max_ 2931, 1715, 1446, 1071 cm^−1^; ^1^H and ^13^C NMR data are given in Table 1 and Table 2, respectively; HRFABMS *m/z* 335.1841 (calcd for C_17_H_28_O_5_Na, 335.1834).

Compound **5**: white amorphous oil; [α]^25^_D_ +42 (*c* = 0.05, MeOH); UV (MeOH) λ_max_ (log ε) 228 (4.20) nm; IR (film) ν_max_ 2929, 1715, 1375, 1075 cm^−1^; ^1^H and ^13^C NMR data are given in Table 1 and Table 2, respectively; HRFABMS *m/z* 333.2036 (calcd for C_18_H_30_O_4_Na, 333.2042).

Compound **6**: white amorphous oil; [α]^25^_D_ −27 (*c* = 0.15, MeOH); UV (MeOH) λ_max_ (log ε) 228 (4.20) nm; IR (film) ν_max_ 2930, 1715, 1457, 1060 cm^−1^; ^1^H and ^13^C NMR data are given in Table 1 and Table 2, respectively; HRFABMS *m/z* 333.2036 (calcd for C_18_H_30_O_4_Na, 333.2042).

**Table 1:** ^1^H NMR spectral data for compounds **1**–**6** in CD_3_OD (500 MHz).

No.	1	2	3	4	5	6
2a	2.46, dd (15.7, 3.9)	2.46 dd (15.7, 3.9)	2.16, dd (16.1, 9.3)	2.33, dd (15.7, 7.8)	2.43 dd (15.9, 4.2)	2.21, dd (15.9, 9.3)
2b	2.83, dd (15.7, 9.3)	2.83, dd (15.7, 9.3)	2.69, dd (16.1, 3.2)	2.39, dd (15.7, 5.4)	2.83, dd (15.9, 9.3)	2.67, dd (15.9, 3.2)
3	4.35, dt (9.3, 3.9)	4.35, dt (9.3, 3.9)	4.07, dt (3.2, 9.3)	4.41, m	4.39, dt (9.3, 4.2)	4.00, dt (3.2, 9.3)
4	2.47, m	2.47, m	1.90, m	1.62, m; 1.73, m	2.38, m	1.83, m
5a	1.35, t (13.5)	1.35, t (13.5)	1.30, m	1.68, m	1.39, m	1.27, m
5b	1.64, dd (13.5, 4.4)	1.64, dd (13,5, 4.4)	1.86, dd (13.0, 4.4)	1.85, m	1.45, dd (13,7, 4.9)	1.77, dd (13.5, 4.4)
7	1.62, m; 1.33, m	1.62, m; 1.35, m	1.61, m; 1.30, m	1.62, m; 1.30, m	1.39, m; 1.35, m	1.87, m; 1.56, m
8	1.31, m	1.32, m	1.31, m	1.31, m	1.34, m	1.31, m
9	1.31, m	1.32, m	1.31, m	1.31, m	1.29, m	1.31, m
10	1.31, m	1.38, m	1.38, m	1.38, m	1.38, m	1.39, m
11	1.31, m	2.05, dt (7.1, 6.7)	2.04, q (7.1)	2.04, q (7.1)	2.04, dt (7.3, 7.1)	2.04, dt (7.3, 6.9)
12	1.31, m	5.50, dd (13.5, 7.1)	5.49, dd (13.9, 7.1)	5.49, dd (14.4, 7.1)	5.49, dd (13.7, 7.3)	5.50, dd (13.9, 7.3)
13	1.97, m	5.97, dd (10.3, 13.5)	5.96, dd (10.5, 13.9)	5.96, dd (10.3, 14.4)	5.96, dd (10.3, 13.7)	5.96, dd (10.3, 13.9)
14	5.40, m	5.99, dd (10.3, 13.3)	5.99, dd (10.5, 13.9)	5.99, dd (10.3, 13.5)	5.99, dd (10.3, 13.7)	5.99, dd (10.3, 13.7)
15	5.40, m	5.55, dq (13.3, 6.6)	5.55, dq (13.9, 6.4)	5.55, dq (13.5, 6.4)	5.55, dq (13.7, 6.6)	5.55, dq (13.7, 6.6)
16	1.62, br s	1.70, d (6.6)	1.69, d (6.4)	1.70, d (6.4)	1.70, d (6.6)	1.70, d (6.6)
17	3.22, s	3.22, s	3.21, s	3.21, s	1.31, s	1.04, s
18	0.85, d (7.1)	0.85, d (7.1)	0.87, d (6.6)		0.88, d (6.9)	0.88, d (6.6)

**Table 2:** ^13^C NMR spectral data for compounds **1**-**8** in CD_3_OD (125 MHz).

No.	1	2	3	4	5	6	7	8
1	175.5, s	175.6, s	174.5, s	174.2, s	173.6, s	174.9, s		
2	32.0, t	32.1, t	36.9, t	39.6, t	32.8, t	37.3, t	148.6, d	145.2, d
3	81.1, d	81.1, d	84.4, d	78.5, d	81.5, d	84.9, d	137.2, s	141.4, s
4	28.1, d	28.1, d	30.8, d	26.2, t	29.3, d	31.4, d	146.0, d	161.9, s
5	35.0, t	35.0, t	40.3, t	31.4, t	37.7, t	43.4, t	128.6, d	129.7, d
6	104.3. s	104.3. s	104.9, s	103.8, s	81.5, s	82.1, s	161.1, s	144.3, d
7	33.7, t	33.7, t	33.6, t	33.6, t	42.0, t	35.9, t	33.6, t	34.9, t
8	23.6. t	23.5, t	23.8, t	23.8, t	23.8, t	24.5, t	28.4, t	31.5, t
9	30.8 *, t	30.3 *, t	30.3, t	30.3, t	30.8, t	30.8, t	30.7 *, t	30.7 *, t
10	30.4 *, t	30.4 *, t	30.4, t	30.4, t	30.4, t	30.6, t	30.6 *, t	30.7 *, t
11	30.1 *, t	33.4, t	33.4, t	33.4, t	33.5, t	33.5, t	30.5 *, t	30.6 *, t
12	30.7 *, t	132.5, d	132.5, d	132.5, d	132.6, d	132.7, d	30.4 *, t	30.5 *, t
13	33.6, t	131.9, d	131.9, d	131.9, d	131.9, d	131.8, d	30.3 *, t	30.5 *, t
14	132.6, d	133.1, d	133.1, d	133.1, d	133.1, d	133.1, d	33.1, t	33.1, t
15	125.7, d	127.4, d	127.4, d	127.4, d	127.3, d	127.3, d	23.7, t	23.7, t
16	18.1, q	18.1, q	18.1, q	18.1, q	18.1, q	18.1, q	14.4, q	14.4, q
17	48.7, q	48.7, q	48.7, q	48.7, q	21.5, q	24.7, q	46.2, q	47.9, q
18	17.1, q	17.1, q	16.9, q		17.4, q	17.4, q	159.5, s	169.5, s

Where * indicates interchangeable signals; s = C; d = CH; t = CH_2_; q = CH_3_.

Compound **7**: white amorphous solid; UV (MeOH) λ_max_ (log ε) 257 (3.32) nm; IR (film) ν_max_ 2918, 1651, 1468, 1346 cm^−1^; ^1^H NMR (CD_3_OD) δ 0.89 (t, 3H, *J* = 6.9 Hz; H-16), 1.25–1.38 (m, 10H), 1.42 (m, 2H, H-10), 1.51 (q, 2H, *J* = 7.8 Hz; H-9), 1.81 (q, 2H, *J* = 7.8 Hz; H-8), 3.11 (t, 2H, *J* = 7.8 Hz; H-7), 4.32 (s, 3H; H-17), 7.96 (d, 1H, *J* = 8.1Hz; H-5), 8.77 (dd, 1H, *J* = 8.1, 1.2 Hz; H-4), 9.12 (d, 1H, *J* = 1.2 Hz; H-2); ^13^C NMR data are given in Table 2; HRFABMS *m/z* 300.1936 (calcd for C_17_H_27_NO_2_Na, 300.1939).

Compound **8**: white amorphous solid; UV (MeOH) λ_max_ (log ε) 258 (3.75) nm; IR (film) ν_max_ 2918, 1652, 1467, 1346 cm^−1^; ^1^H NMR (CD_3_OD) δ 0.89 (t, 3H, *J* = 6.9 Hz; H-16), 1.25–1.36 (m, 12H), 1.40 (m, 2H, H-9), 1.70 (q, 2H, *J* = 7.8 Hz; H-8), 3.16 (t, 2H, *J* = 7.8 Hz; H-7), 4.30 (s, 3H; H-17), 7.84 (d, 1H, *J* = 6.4 Hz; H-5), 8.58 (dd, 1H, *J* = 6.4, 1.2 Hz; H-6), 8.80 (d, 1H, *J* = 1.2 Hz; H-2); ^13^C NMR data are given in Table 2; HRFABMS *m/z* 300.1934 (calcd for C_17_H_27_NO_5_Na, 300.1939).

### 3.4. MTPA Reaction of Reduced Compounds ***1*** and ***4***

Compounds **1** and **4** were first converted into their methyl ester with diazomethane in MeOH and then the cyclic peroxy group of each compound was reduced into its keto alcohol by treatment with Zn and acetic acid in ether as reported in a paper [14]. To a stirred solution of reduced compound **1** (2.0 mg) and dried pyridine (20 µL) in dry CH_2_Cl_2_ (0.5 mL) at room temperature, *R*-MTPA-Cl (15 µL) was added. The reaction progress was monitored by TLC chromatography on silica gel (Hex:EtOAc = 4:1). After ~4 h, the reaction mixture was quenched by the addition of H_2_O and dimethyl ether. The organic layer was concentrated *in vacuo*. The crude product mixture was eluted by silica-gel SPE with hexane: ethyl acetate (5:1) to give the *S*-MTPA-ester reduced **1*S*** as a pale yellow gum. ^1^H NMR (CDCl_3_, 500 MHz) δ 0.88 (3H, d, *J* = 6.9 Hz; H-18), 2.19 (m, 1H; H-5a), 2.43 (m, 1H; H-5b), 2.47 (m, 1H; H-4), 2.54 (dd, 1H, *J* = 16.1, 3.4 Hz; H-2a), 2.65 (dd, 1H *J* = 16.1, 9.2 Hz; H-2b), 3.61 (s, 3H; OCH_3_), 5.53 (m, 1H; H-3).

In an entirely analogous way, the *R*-MTPA-ester reduced **1*R*** was obtained using *S*-MTPA-Cl. ^1^H NMR (CDCl_3_, 500 MHz) δ 0.84 (d, 3H, *J* = 6.9 Hz; H-18), 2.07 (m, 1H; H-5a), 2.29 (m, 1H; H-5b), 2.43 (m, 1H; H-4), 2.56 (dd, 1H, *J* = 16.1, 3.9 Hz; H-2a), 2.67 (dd, 1H, *J* = 16.1, 9.3 Hz; H-2b), 3.66 (s, 3H; OCH_3_), 5.50 (m, 1H; H-3).

The same method was applied to the reduced compound **4** to afford the *S*-MTPA-ester reduced **4*S*** ^1^H NMR (CDCl_3_, 500 MHz) δ 1.83 (m, 1H; H-4a), 2.01 (m, 1H; H-4b), 2.26 (t, 2H, *J* = 7.1 Hz; H-7), 2.34 (m, 2H; H-5), 2.61 (dd, 1H, *J* = 15.9, 4.9 Hz; H-2a), 2.70 (dd, 1H, *J* = 15.9, 7.8 Hz; H-2b), 3.66 (s, 3H; OCH_3_), 5.46 (m, 1H; H-3) and *R*-MTPA-ester reduced **4***R* ^1^H NMR (CDCl_3_, 500 MHz) δ 1.92 (m, 1H; H-4a), 2.05 (m, 1H; H-4b), 2.34 (t, 2H, *J* = 7.3 Hz; H-7), 2.43 (m, 2H; H-5), 2.59 (dd, 1H, *J* = 16.1, 4.9 Hz; H-2a), 2.67 (dd, 1H, *J* = 16.1, 8.1 Hz; H-2b), 3.59 (s, 3H; OCH_3_), 5.48 (m, 1H; H-3).

### 3.5. Biological Activities

Cytotoxicity was examined by an MTT assay. After RAW264.7 aliquots were seeded (3 × 10^5^) in microplate wells. The compounds (1, 5 and 10 µg/mL) were added to each well and incubated with 10 µL of an MTT solution (5 mg/mL) for 4 h at 37 °C under 5% CO_2_ and 95% air. Then the supernatant was removed and the formazan crystals were dissolved by the addition of 100 µL of DMSO. An automatic microplate reader was used to read the absorbance of each well at 590 nm. Compound **1** exhibited an EC_50_ value of 4.5 µg/mL, 6.1 µg/mL for **2**, 4.9 µg/mL for **3**, 5.2 µg/mL for **4**, 0.2 µg/mL for **5**, 3.1 µg/mL for **6**.

The antifungal activity of all isolated compounds was tested against the fungus *C. albicans* (ATCC10231). The fungus was grown in ATCC790 media (0.5% D-glucose, 0.1% yeast extract, 0.1% peptone, and 3% sea water) at 25 °C for 2 days. After incubation, 100 µL fungus was smeared on an ATCC790 agar plate and on it paper discs were placed to spot compounds. The activity degree of agar-diffusible compounds was estimated by measuring the diameter of inhibition zone. Compound **1** showed an inhibition zone of 21 mm, 11 mm for **2**, 16 mm for **3**, 16 mm for **4**, 17 mm for **5**, and 14 mm for **6**, compared with 15 mm for amphotericin used as positive control. Compounds **7** and **8** did not exhibit activities for the two tests.

## 4. Conclusions

Chemical studies from the sponge *P. simplex*, collected from Keomun Island, South Korea, led to the isolation of six new cyclic peroxide polyketides and two new pyridinium alkaloids with a straight decane or decane-derived chain. Compounds **2**–**6** possessed the same side chain, but differed in substituents or stereocenters within a 1,2-dioxane ring. In particular, the isolation of secondary metabolites with methoxy or methyl group on C-6 from the same extract was biosynthetically attractive. Compounds **1**–**6** exhibited cytotoxic effects toward RAW264.7 cells and strong activity against *C*. *albicans*. Considering the strong cytotoxicity of **1**–**6**, we could not conclude that the activity against *C. albicans* was specifically antifungal. Two unrelated compounds **7** and **8** contained a trigonelline skeleton that varied in the position of a decane side chain. In this study, these latter compounds did not exhibit cytotoxicity and antifungal activities.

## Supplementary Files

Supplementary File 1 Supplementary Information (PDF, 218 KB)
