# Supplementary material for: New Constituents from the Korean Sponge Plakortis simplex
Source: Mar Drugs. 2013 Nov 5;11(11):4407–18. doi: 10.3390/md11114407 (PMC3853735; doi:10.3390/md11114407)

Supplementary Information

Figure S1. ^{1}H NMR spectrum of **1** in CD_3OD at 500MHz. 2
Figure S2. 13C NMR spectrum of **1** in CD_3OD at 125MHz. 3
Figure S3. ^{1}H NMR spectrum of **2** in CD_3OD at 500MHz. 4
Figure S4. ^{13}C NMR spectrum of **2** in CD_3OD at 125MHz. 5
Figure S5. ^{1}H NMR spectrum of **3** in CD_3OD at 500MHz. 6
Figure S6. ^{13}C NMR spectrum of **3** in CD_3OD at 125MHz. 7
Figure S7. ^{1}H NMR spectrum of **4** in CD_3OD at 500MHz. 8
Figure S8. ^{13}C NMR spectrum of **4** in CD_3OD at 125MHz. 9
Figure S9. ^{1}H NMR spectrum of **5** in CD_3OD at 500MHz. 10
Figure S10. ^{13}C NMR spectrum of **5** in CD_3OD at 125MHz. 11
Figure S11. ^{1}H NMR spectrum of **6** in CD_3OD at 500MHz. 12
Figure S12. ^{13}C NMR spectrum of **6** in CD_3OD at 125MHz. 13
Figure S13. ^{1}H NMR spectrum of **7** in CD_3OD at 500MHz. 14
Figure S14. ^{13}C NMR spectrum of **7** in CD_3OD at 125MHz. 15
Figure S15. ^{1}H NMR spectrum of **8** in CD_3OD at 500MHz. 16
Figure S16. ^{13}C NMR spectrum of **8** in CD_3OD at 125MHz. 17

Figure S1. ^{1}H NMR spectrum of **1** in CD_3OD at 500MHz.

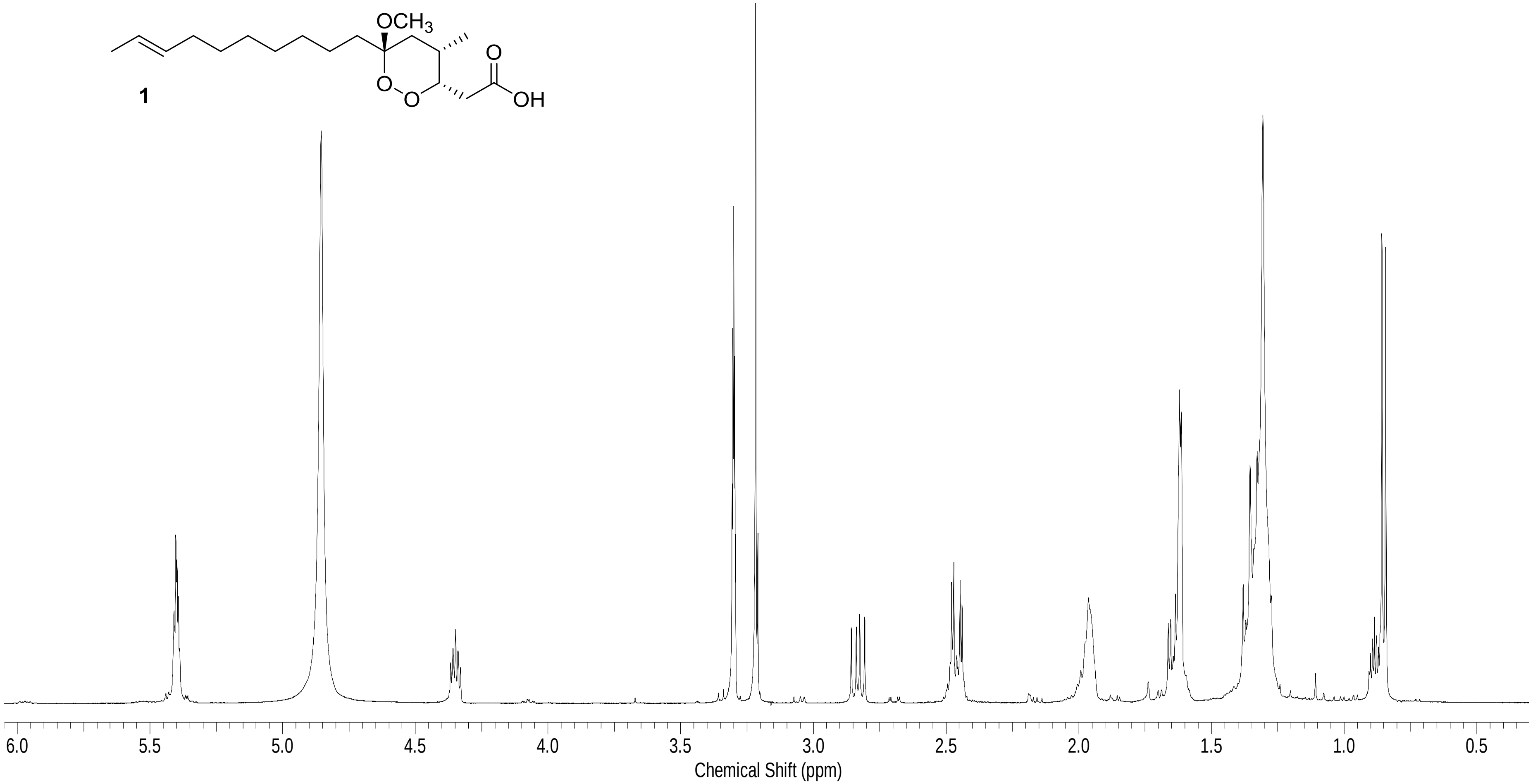

Figure S2. ^{13}C NMR spectrum of **1** in CD_3OD at 125MHz.

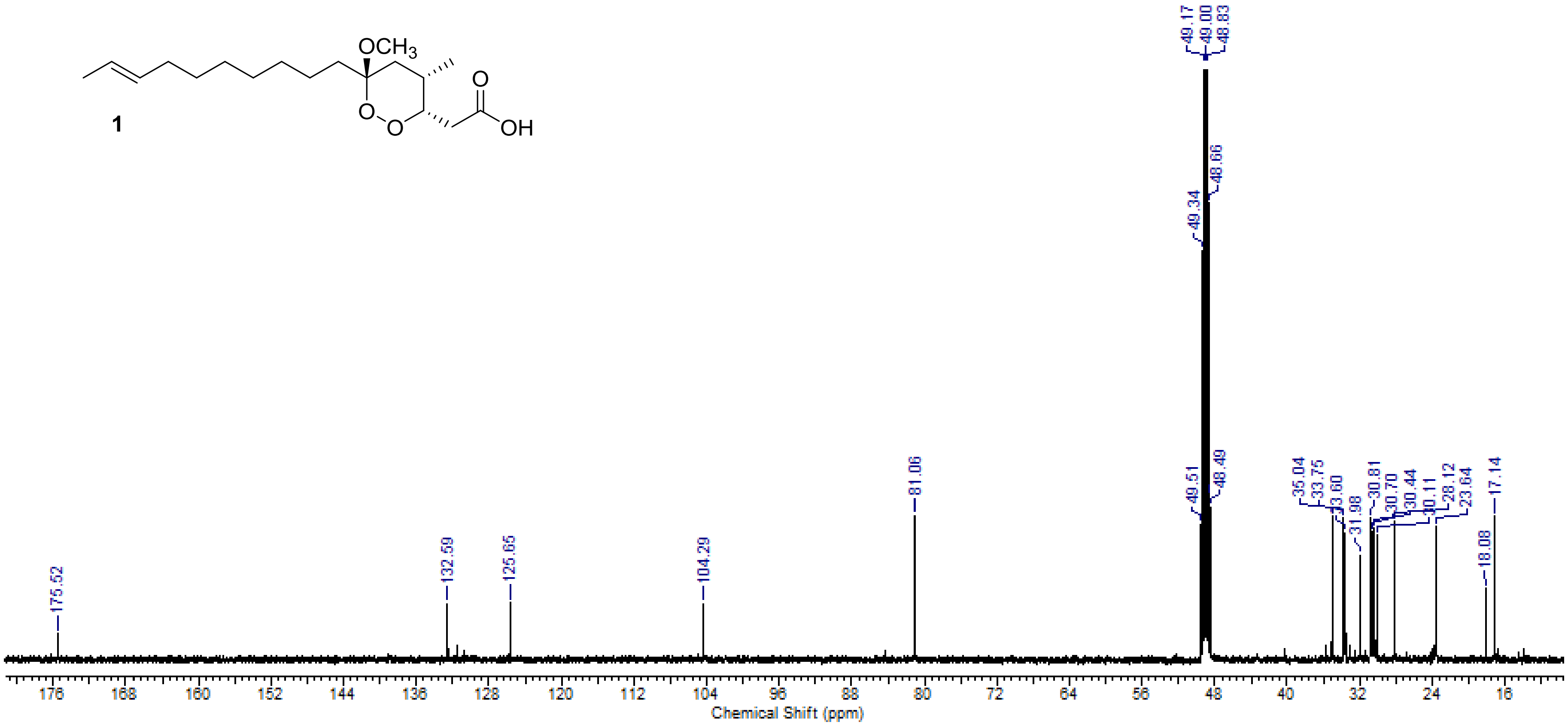

Figure S3. ^{1}H NMR spectrum of **2** in CD_3OD at 500MHz.

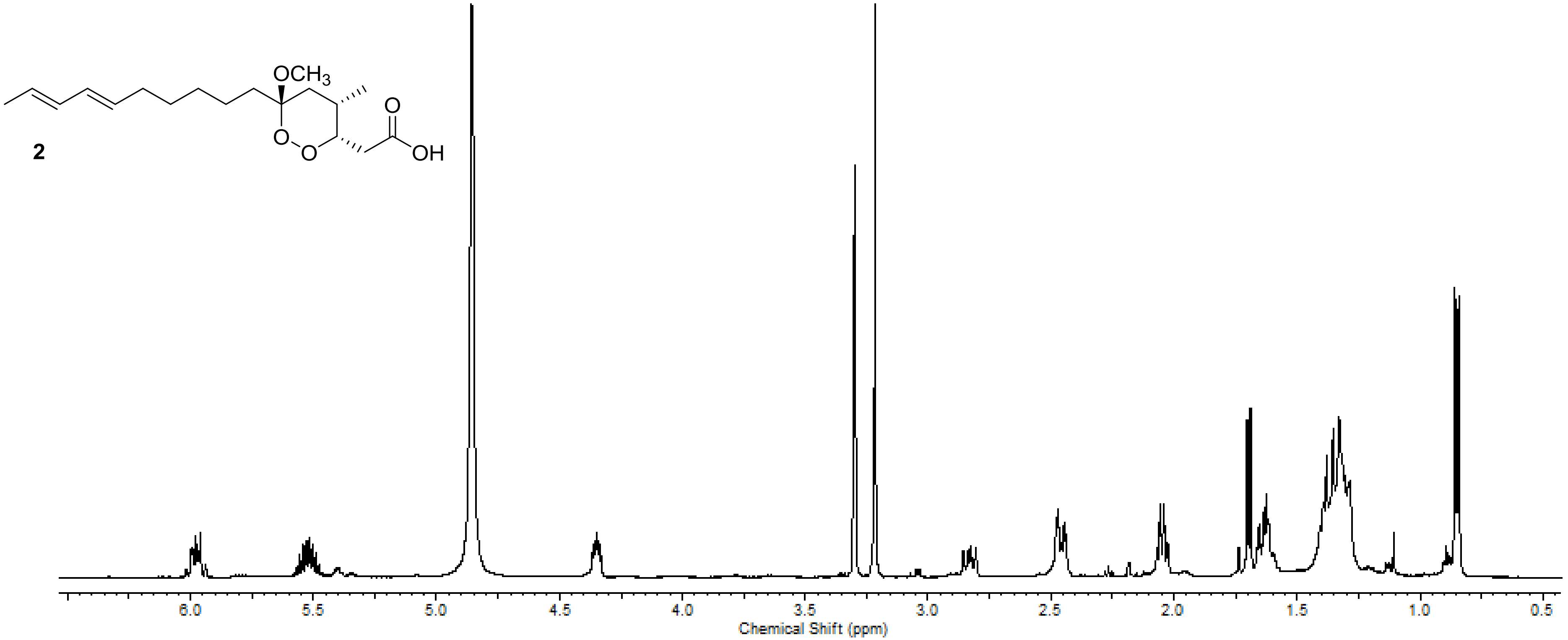

Figure S4. ^{13}C NMR spectrum of **2** in CD_3OD at 125MHz.

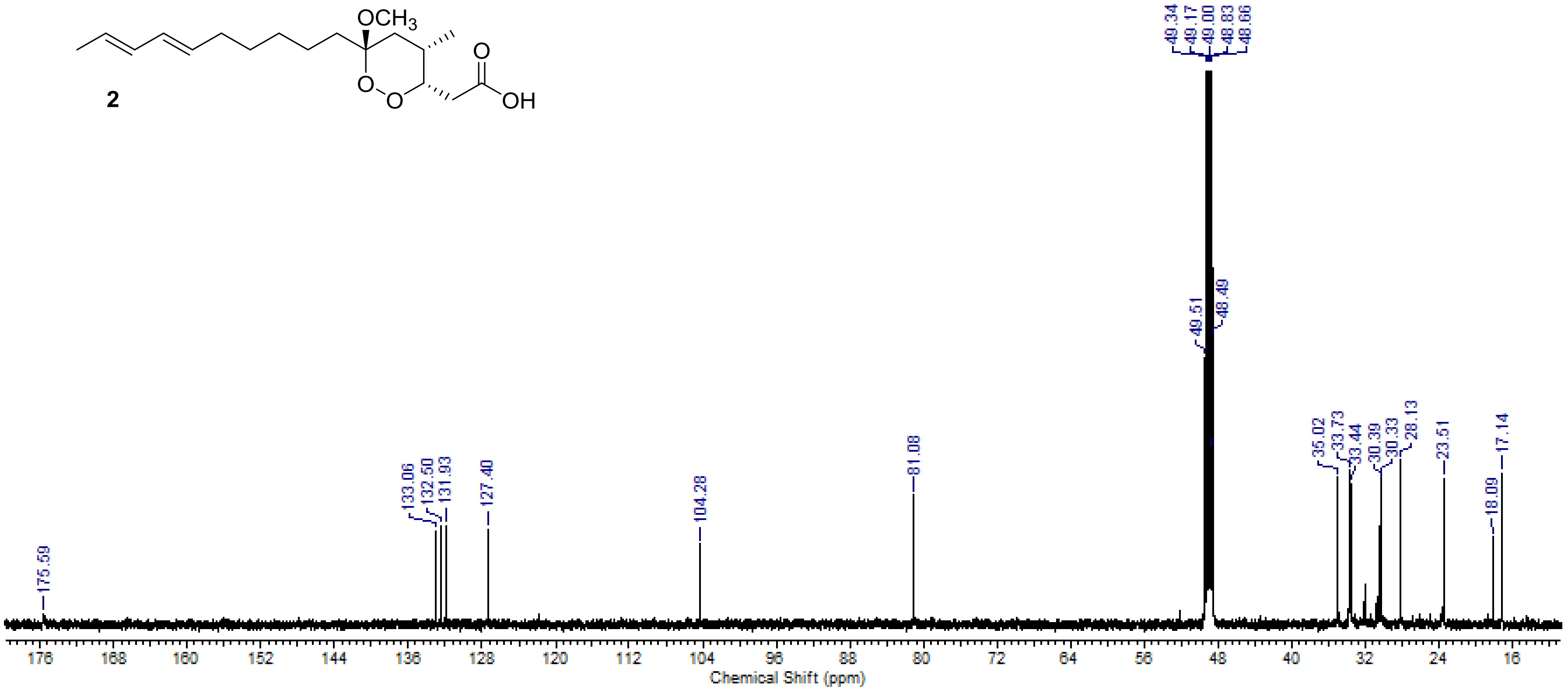

Figure S5. ^{1}H NMR spectrum of **3** in CD_3OD at 500MHz.

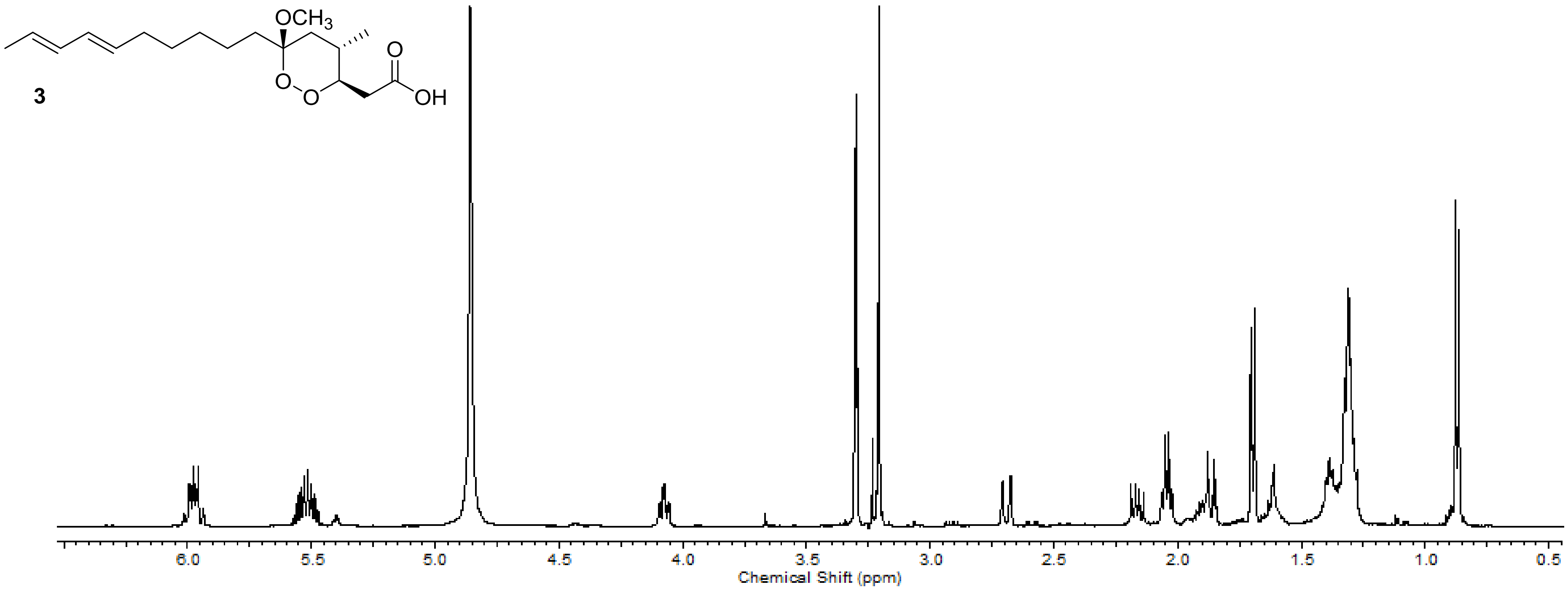

Figure S6. ^{13}C NMR spectrum of **3** in CD_3OD at 125MHz.

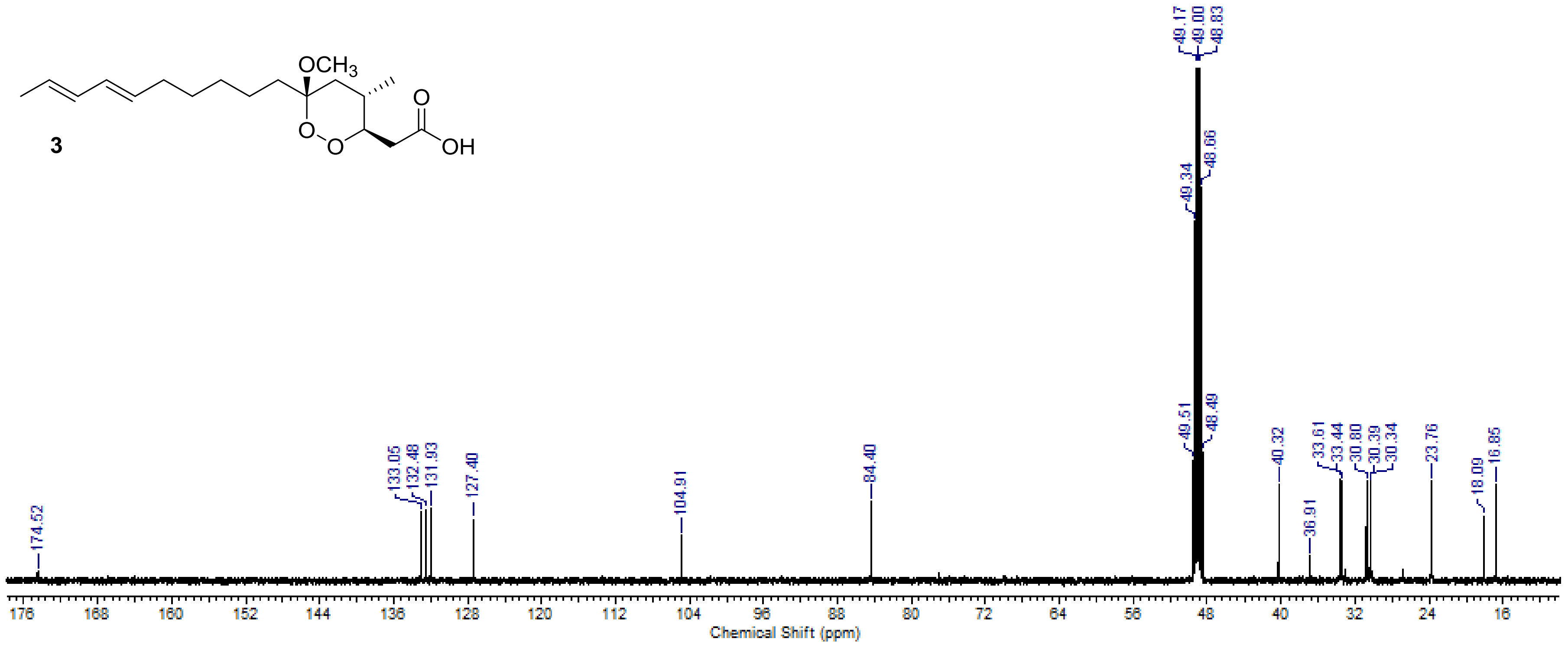

Figure S7. ^{1}H NMR spectrum of **4** in CD_3OD at 500MHz.

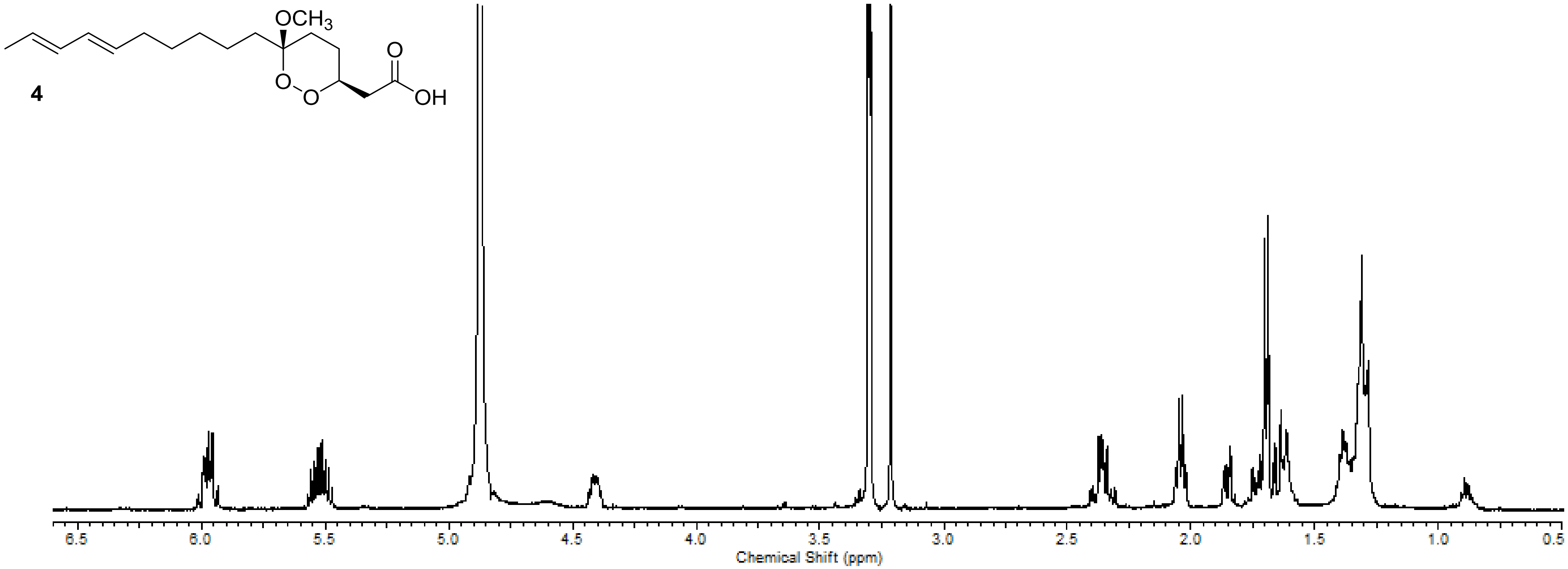

Figure S8. ^{13}C NMR spectrum of **4** in CD_3OD at 125MHz.

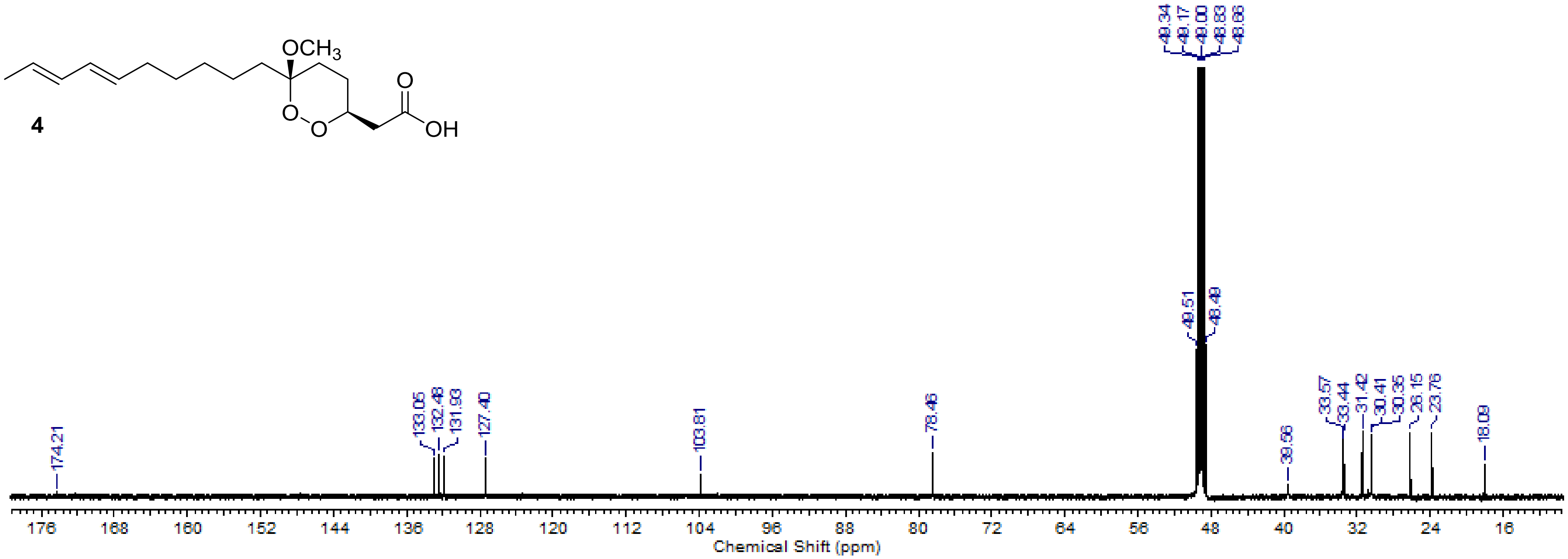

Figure S9. ^{1}H NMR spectrum of **5** in CD_3OD at 500MHz.

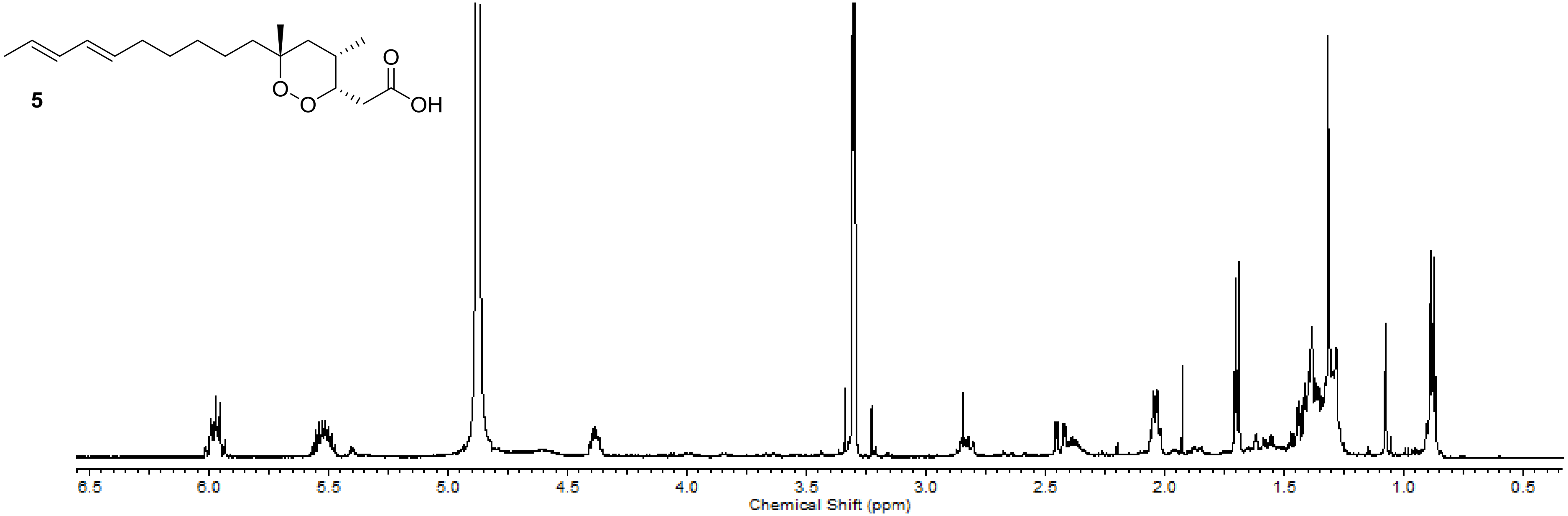

Figure S10. ^{13}C NMR spectrum of **5** in CD_3OD at 125MHz.

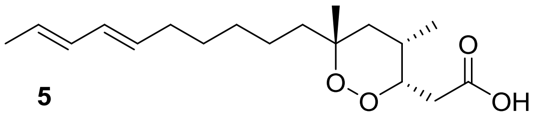

5

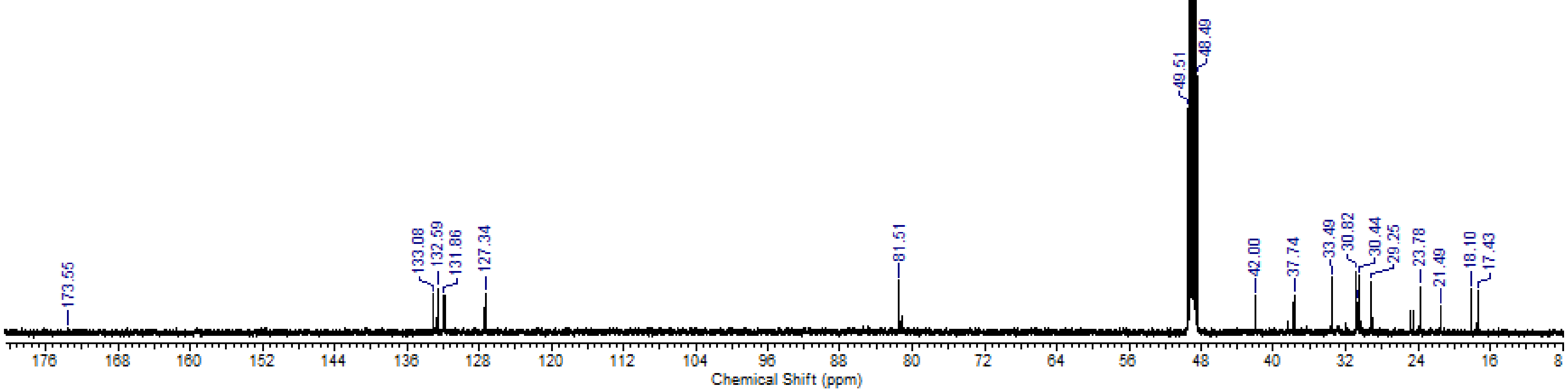

Figure S11. ^{1}H NMR spectrum of **6** in CD_3OD at 500MHz.

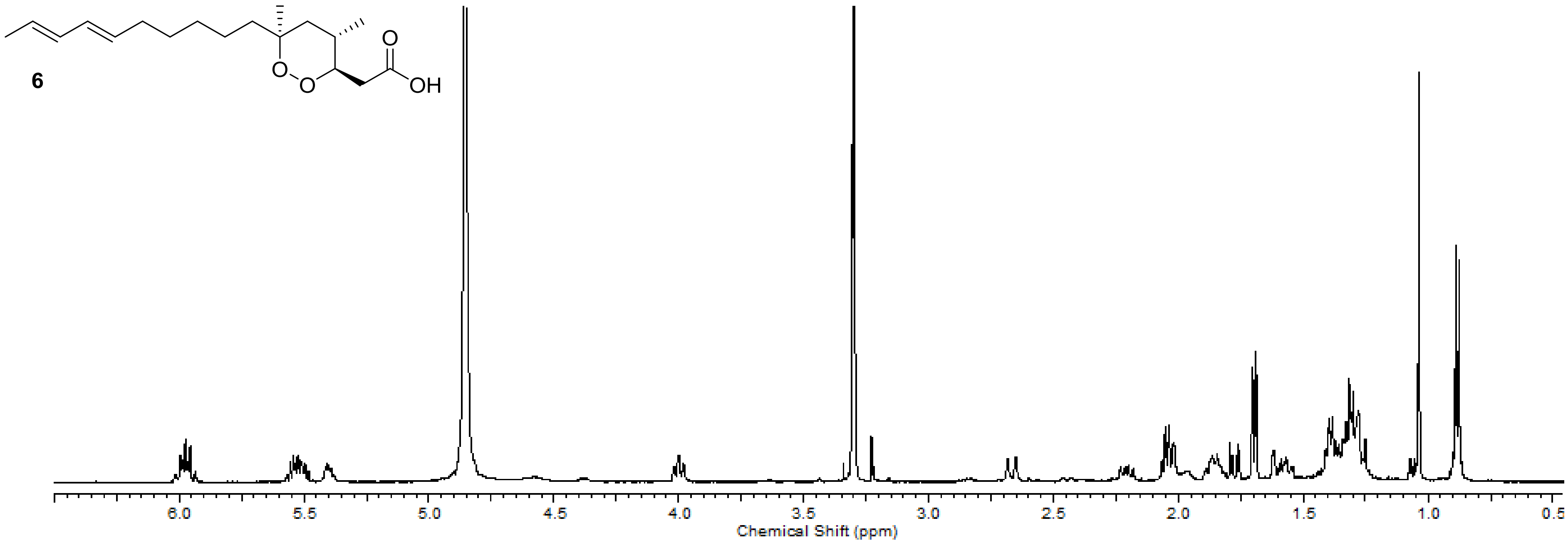

Figure S12. ^{13}C NMR spectrum of **6** in CD_3OD at 125MHz.

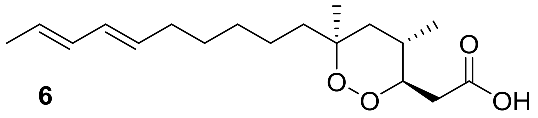

6

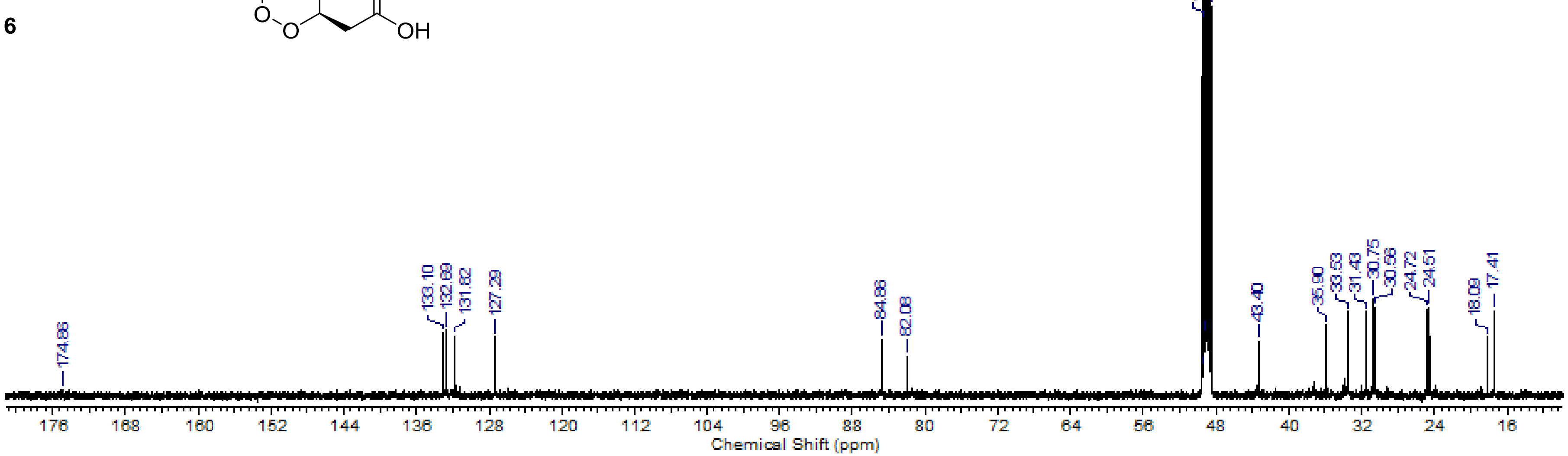

Figure S13. ^{1}H NMR spectrum of **7** in CD_3OD at 500MHz.

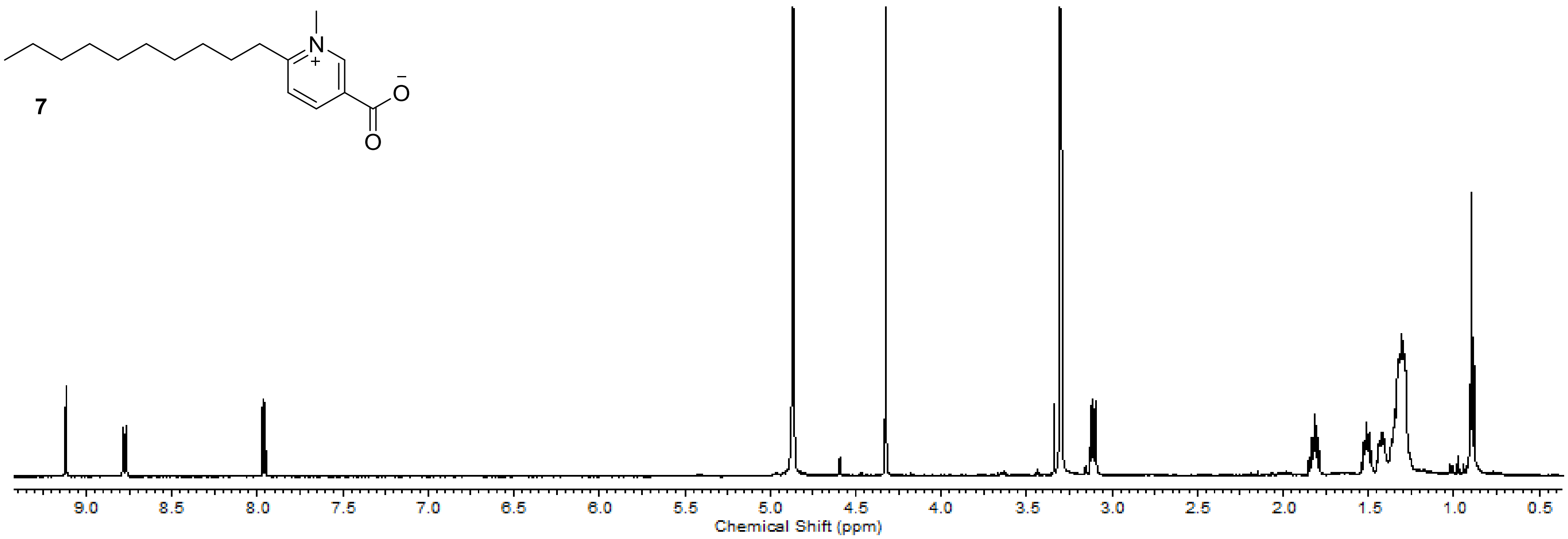

Figure S14. ^{13}C NMR spectrum of **7** in CD_3OD at 125MHz.

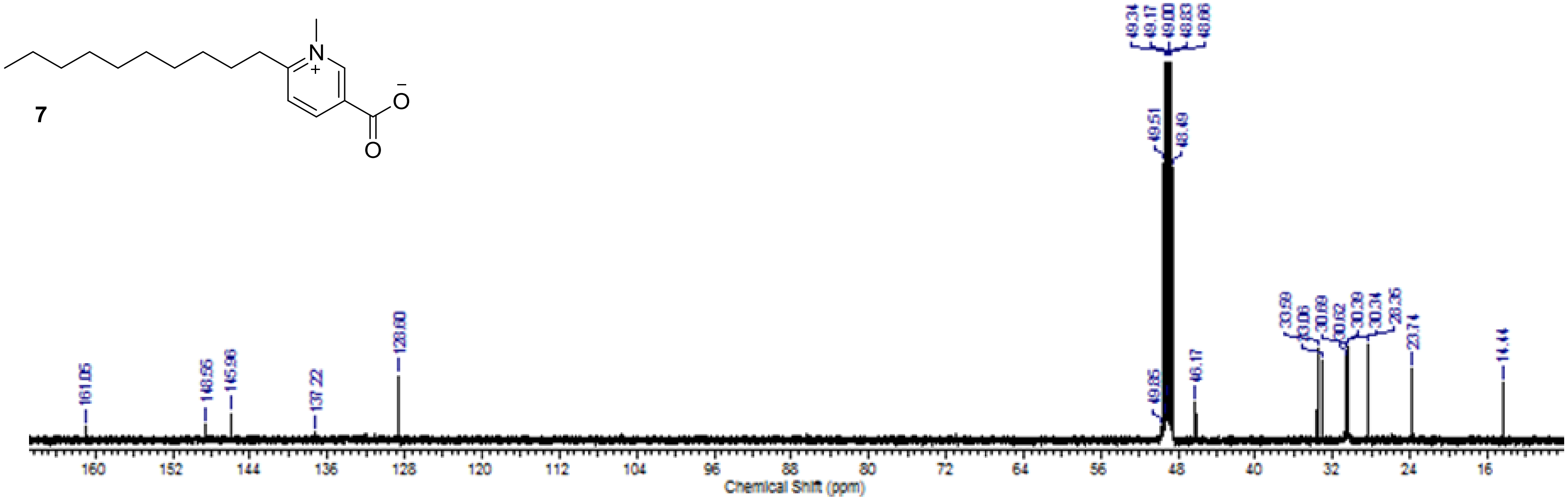

Figure S15. ^{1}H NMR spectrum of **8** in CD_3OD at 500MHz.

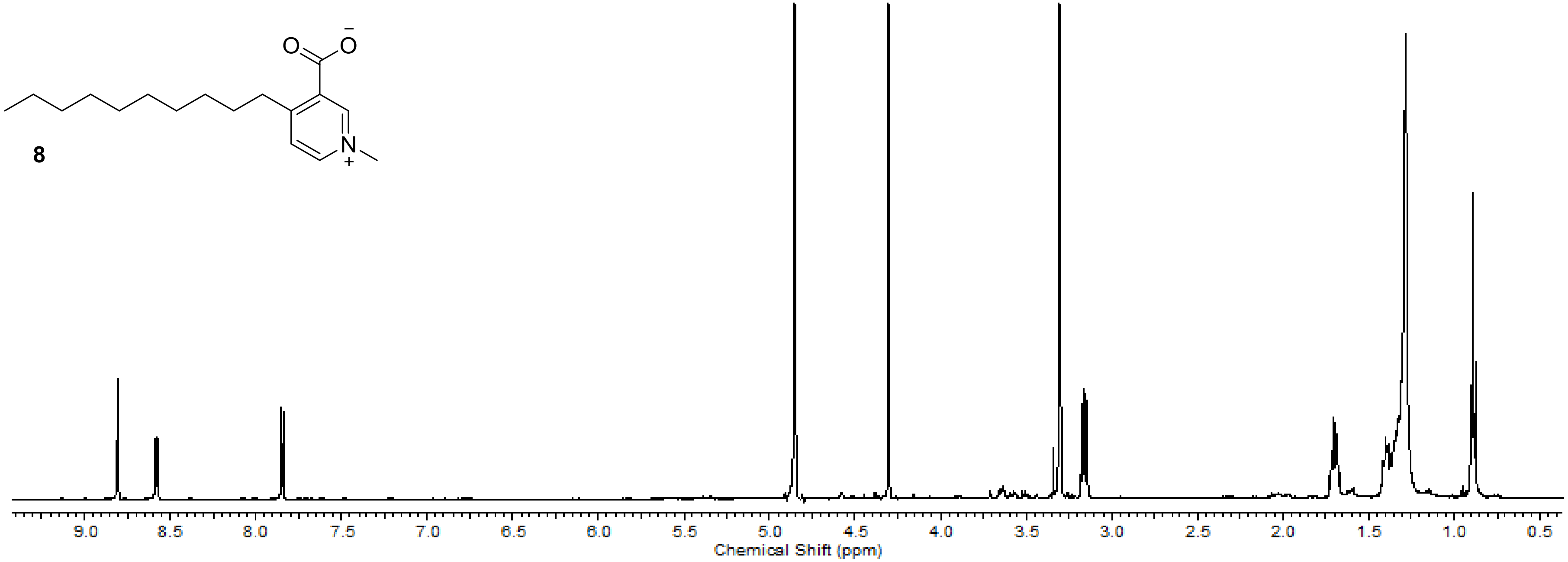

Figure S16. ^{13}C NMR spectrum of **8** in CD_3OD at 125MHz.

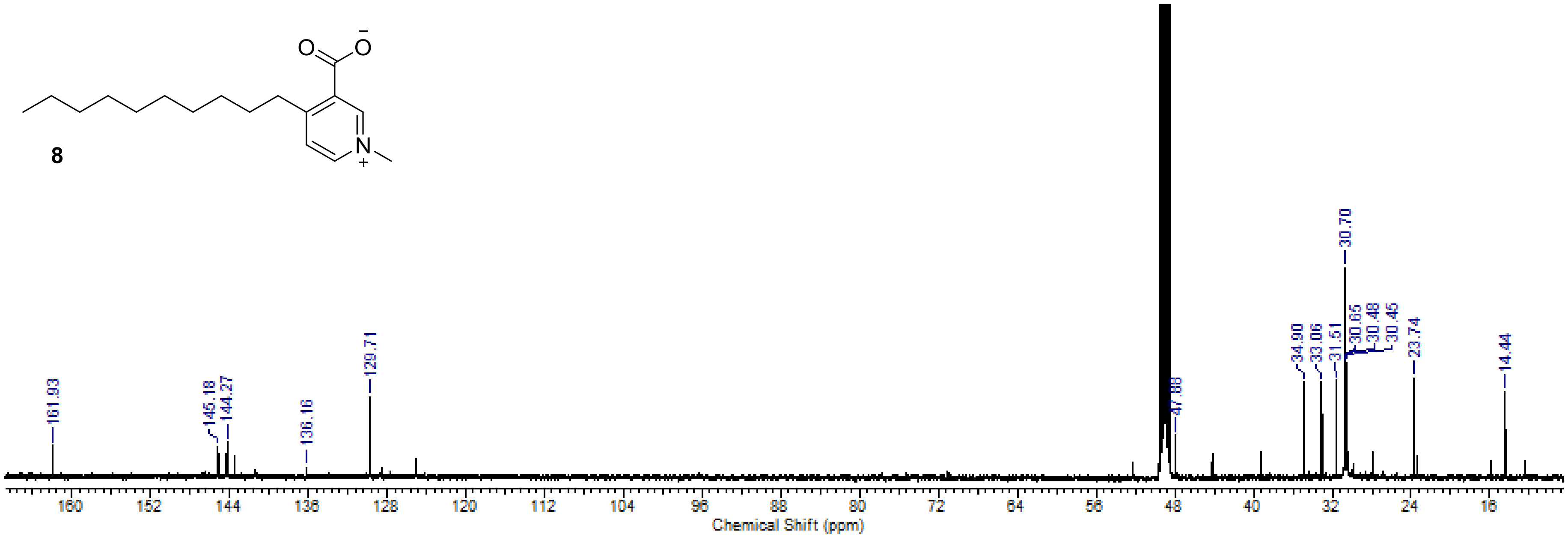

Supplement: Supplementary File 1 — Supplementary Information (PDF, 218 KB) [file marinedrugs-11-04407-s001.pdf]
